# Region specific knockdown of Parvalbumin or Somatostatin produces neuronal and behavioral deficits consistent with those observed in schizophrenia

**DOI:** 10.1038/s41398-019-0603-6

**Published:** 2019-10-21

**Authors:** Stephanie M. Perez, Angela Boley, Daniel J. Lodge

**Affiliations:** UT Health San Antonio, Department of Pharmacology, Center for Biomedical Neuroscience, 7703 Floyd Curl Drive, MC 7764, San Antonio, TX 78229 USA

**Keywords:** Neuroscience, Hippocampus

## Abstract

The anterior hippocampus and prefrontal cortex are regions linked to symptoms of schizophrenia. The anterior hippocampus is believed to be a key regulator of the mesolimbic dopamine system and is thought to be the driving force contributing to positive symptoms, while the prefrontal cortex is involved in cognitive flexibility and negative symptoms. Aberrant activity in these regions is associated with decreases in GABAergic markers, indicative of an interneuron dysfunction. Specifically, selective decreases are observed in interneurons that contain parvalbumin (PV) or somatostatin (SST). Here, we used viral knockdown in rodents to recapitulate this finding and examine the region-specific roles of PV and SST on neuronal activity and behaviors associated with positive, negative and cognitive symptoms. We found that PV and SST had differential effects on neuronal activity and behavior when knocked down in the ventral hippocampus (vHipp) or medial prefrontal cortex (mPFC). Specifically, SST or PV knockdown in the vHipp increased pyramidal cell activity of the region and produced downstream effects on dopamine neuron activity in the ventral tegmental area (VTA). In contrast, mPFC knockdown did not affect the activity of VTA dopamine neuron activity; however, it did produce deficits in negative (social interaction) and cognitive (reversal learning) domains. Taken together, decreases in PV and/or SST were sufficient to produce schizophrenia-like deficits that were dependent on the region targeted.

## Introduction

A prominent pathology observed in individuals with schizophrenia are deficits in GABAergic neurotransmission^1–4^. GABAergic interneurons modulate neuronal circuitry, balance neuronal excitation and inhibition and establish network oscillations^5–7^. Interneurons of the hippocampus and frontal cortex are largely comprised of independent subpopulations including those that express the calcium binding protein, parvalbumin (PV) or the neuropeptide, somatostatin (SST)^8^. PV-containing interneurons are fast-spiking and target the axon initial segment and cell body to regulate pyramidal cell firing. Conversely, SST-containing interneurons exhibit a slow firing pattern and synapse on distal dendrites of pyramidal cell. A selective loss of PV and SST expression in hippocampal regions and frontal cortex have been observed post-mortem in brain tissue harvested from patients^4,9–11^. Further, GABAergic interneuron deficits have also been observed in analogous regions (ventral hippocampus and prefrontal cortex) in rodent models;^12–14^ however, whether this contributes to, or is a cause of the disease has not been conclusively demonstrated.

Mesolimbic dopamine system dysfunction has been linked to the positive symptoms of the disease;^15,16^ however, there is a lack of evidence suggesting a primary pathology in the dopamine system itself^17^. Alternatively, it has been suggested that it is the regulation of the mesolimbic dopamine system by the ventral hippocampus (vHipp) that is dysfunctional in schizophrenia. Individuals with schizophrenia display hyperactivity in hippocampal regions at rest^18^, which is also present in rodent models of the disease^19^. We have previously demonstrated that a selective decrease in PV expression, in the vHipp, results in aberrant dopamine system function^20–22^. Further, normal dopamine system function was restored in rodent models of the disease following the transplantation of PV or SST interneuron precursors into the vHipp^21,22^. However, PV and SST neurons produced different effects on discrete behavioral domains, with PV interneuron transplants reversing deficits in dopamine system function, social withdrawal and two different measures of cognitive flexibility^22^. In contrast, SST transplants were able to reverse only one of these behavioral alterations, reversal learning^22^.

Taken together, evidence suggests that a deficit in intrinsic GABAergic signaling may contribute to the pathophysiology of schizophrenia; however, the relative contributions of PV and SST neurons throughout different brain regions is yet to be established. Here we recapitulate the pathological finding of decreased interneuron function by lentiviral delivered shRNA to knockdown PV or SST in the vHipp or medial prefrontal cortex (mPFC) in both male and female rats. We demonstrate that this produces alterations in key brain circuits which translates to behaviors that correlate with the positive, negative and cognitive deficits observed in schizophrenia. These findings support the notion that aberrant interneuron function may be a key pathology in schizophrenia and that targeting this system may be a better therapeutic approach.

## Materials and methods

All experiments were performed in accordance with the guidelines outlined in the USPH Guide for the Care and Use of Laboratory Animals and were approached by the Institutional Animal Care and the Use Committee of the UT Health San Antonio. Male and female rats were used in this study and both pooled and disaggregated data are presented in the results.

### Viral shRNA-mediated gene knockdown

All survival surgical procedures were performed under general anesthesia in a semi-sterile environment. Male (225–250 g) and female (180–225 g) Sprague–Dawley rats were anesthetized with Fluriso™ (2–5% Isoflurane, USP with oxygen flow at 1 L/min) and placed in a stereotaxic apparatus (Kopf, Tujunga, CA) using blunt atraumatic ear bars and a core body temperature of 37 °C was maintained. Rats from various liters were randomly selected and were bilaterally injected (0.75 µL/side) with high-titer lentivirus particles containing GIPZ vectors (Dharmacon; Lafayette, CO) expressing a GFP reporter and shRNA targeting either PV (mature antisense: TAGCAGACAAGTCTCTGGC), SST (mature antisense: AGAAGTACTTGGCCAGTTC) or a non-silencing control, into either the vHipp (A/P: −4.8 mm from bregma; M/L: ± 4.8 mm from midline; D/V: −7.5 mm ventral of skull surface) or mPFC (A/P: + 3.0 mm from bregma; M/L: ± 0.6 mm from midline; D/V: −4.5 mm ventral of skull surface). Rats received postoperative ketoprofen (5 mg/kg, s.c.) and were housed under ABSL 2 conditions for 72 h before being transferred to standard housing conditions. Rats were housed for 6 weeks prior to behavioral and electrophysiological experiments to allow for sufficient viral expression. Sample sizes were based on power analysis using previously published data with the same assays and paradigms. No animals were excluded from the current study.

### In vivo electrophysiology

For non-survival surgery, chloral hydrate (400 mg/kg; i.p.) was used to anesthetize rats prior to placement in a stereotaxic apparatus (Kopf; Tujunga, CA). A core body temperature of 37 °C was maintained, while supplemental anesthesia was administered as required to maintain compression of limb withdrawal reflex. Extracellular glass microelectrodes (impedance 6–10 MΩ) were lowered into the vHipp (A/P: −5.0 mm from Bregma; M/L: ± 4.8 mm from Bregma; D/V: −4.0 to −8.5 mm ventral of the brain surface), mPFC (A/P: + 3.0 mm from Bregma; M/L: ± 0.6 mm from Bregma; D/V: −3.0 to −5.0 mm ventral of the brain surface) or VTA (A/P: −5.3 mm from Bregma; M/L: ± 0.6 mm from Bregma; D/V: −6.5 to −9.0 mm ventral of the brain surface). The firing frequency of spontaneously active putative pyramidal neurons in the vHipp and mPFC were measured and identified as previously published for each respective region (vHipp: neurons with firing frequencies <2 Hz;^20,21,23^ mPFC: action potential duration between 0.7 and 2 ms and firing frequencies <14 Hz^24^). Spontaneously active dopamine neurons were identified using previously established criteria: (1) action potential duration >2 ms (2) frequency between 0.5 and 15 Hz. Three parameters of dopamine activity were measured: (1) population activity (the number of spontaneously active dopamine neurons encountered per track) (2) basal firing rate (3) the proportion of action potentials occurring in bursts. Electrophysiological recordings were analyzed by one-way ANOVA, followed by a Holm–Sidak post-hoc test.

### Stimulant-induced locomotion

Rats were placed in an open field arena (Med Associates, VT, USA) and spontaneous locomotor activity in the *x–y* plane was determined for 30 min by beam breaks and recorded with Open Field Activity software (Med Associates). Following a 30 min baseline recording, all rats were injected with increasing doses of D-amphetamine sulfate (0.5 mg/kg and 2.0 mg/kg, *i.p*.). Locomotor activity was recorded for an additional 30 min immediately following each dose. Locomotor data were analyzed by separate two-way ANOVA’s (treatment × time), one for each of the relevant drug periods (baseline, 0.5 mg/kg and 2.0 mg/kg) followed by a Holm–Sidak post-hoc test.

### Social interaction (SI)

SI was performed as described previously^25^. Briefly, rats were placed individually in a testing arena (100 × 100 × 40 cm^3^) for 5 min per day for 2 days prior to testing. On the test day, experimental rats were placed in the arena with a weight-matched “stimulus” rat. The 5 min test was recorded by video camera for offline analysis by two separate blind experimenters. The time the test animal spent actively engaged in social interaction (i.e., sniffing, climbing on, following, grooming, or wrestling) with the stimulus rat was measured. The experimenter was blinded to the treatment of the group while scoring the time spent interacting. SI data was analyzed by one-way ANOVA, followed by a Holm–Sidak post hoc test.

### Attentional Set-Shifting (AST)

Rats were examined for cognitive flexibility using the attentional set-shifting task (adapted from^26^). A rectangular arena divided into three quadrants was used for the testing arena. One quadrant was marked as the start box, while the remaining quadrants contained pots defined by a pair of cues along two stimulus dimensions: odor and digging medium. Rats were food restricted (80% of normal dietary intake) for a minimum of seven days prior to the testing day. A ‘Cheerio’ (toasted whole grain cereal) reward was placed at the bottom of the “positive” pot and buried in the digging medium. Rats were then trained to reliably dig in pots to obtain the reward during the habituation period. The following day, rats underwent training in which they were taught simple discrimination tasks, to reach a criterion of six consecutive correct trials. On the day of testing, rats were exposed to a series of tasks of increasing difficulty that included a compound discrimination (CD), an intra-dimensional shift (ID), two reversals (R1 and R2) and an extra-dimensional shift (ED). The testing portion of AST typically took 3–8 h to complete per rat. Based on our previously published work^27^, and that of others^28^, we anticipated deficits in reversal learning (R1) and ED, that were analyzed by one-way ANOVA, followed by a Holm–Sidak post hoc test. The experimenter was blinded to the treatment of the subject during all portions of AST.

### Western Blot

Protein expression of GAD 65/67 was measured using western blot. The vHipp/mPFC was dissected from a subset of control and PV/SST knockdown rats (*n* = 6–12 rats per group) and homogenized in ice cold homogenization buffer (750 mL for vHipp and 500 mL for mPFC) containing a protease inhibitor (cOmplete™, Mini, EDTA-free Protease Inhibitor Cocktail; Sigma; 11836170001). Samples were4 centrifuged (14,000 r.p.m. for 2 min) and the supernatant containing protein fractions was collected. Protein concentrations were determined using the Bradford method before incubation with Laemmli Sample Buffer containing 5% dithiothreitol (10 min at 90 °C) and separated at 200 mA on a 10% acrylamide gel (Any kD™ Mini-PROTEAN® TGX™ Precast Protein Gels; BioRad). Proteins were transferred to a PVDF membrane (Trans-Blot®Turbo™ Mini PVDF Transfer Pack; BioRad) using a Trans-Blot Turbo System (BioRad; Hercules, CA, USA). Membranes were then washed 3 times (10 min each) in TBST and blocked (30 min) in 5% BSA in TBST before incubation in primary antibody for GAD65/67 (Anti-GAD56 + GAD67 antibody; 1:15,000) for 1 h at 4 °C. Membranes were washed 3 times (10 min in TBST), followed by incubation in secondary antibody (Goat Anti-Rabbit; 1:10,000) at room temperature (1 h). Next, membranes were treated with Pierce™ ECL Western Blotting Substrate (1 min) and protein signal was captured using a G:BOX-XT4 Chemi system (Syngene) or exposed to high-performance chemiluminescence film (Amersham Hyperfilm ECL). Membranes were then washed 3 times (10 min) in TBST and stripped with Restore™ Western Blot Stripping Buffer (15 min) to allow for re-probing with GAPDH (Anti-GAPDH antibody; 1:1000 and Goat Anti-Mouse; 1:5000). Western blot films were scanned, and optical density was measured using ImageJ.

### Histology

**Fig. 1:**
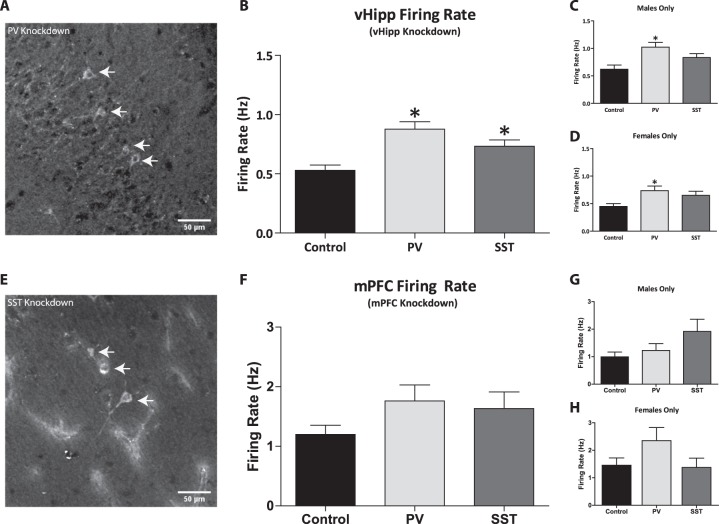
Selective knock down of PV or SST expression in the ventral hippocampus caused a significant increase in the average firing rate of putative pyramidal neurons. Representative sections displaying lentiviral transfected cells expressing shRNA targeting parvalbumin (PV, **a**) or somatostatin (SST, **e**). Selective knock down of PV or SST expression in the ventral hippocampus (vHipp), of male and female rats combined, is sufficient to cause a significant increase in the average firing rate of putative pyramidal neurons **b**. Further analysis, of male **c** and female **d** populations individually, reveals that decreases in the expression of PV caused a significant increase in the average firing rate of vHipp putative pyramidal neurons. *n* = 39–88 cells per group. *P < 0.05. Conversely, selective regional knock down of PV or SST expression in the medial prefrontal cortex (mPFC) of both male and female rats had no significant effect on the average firing rate of putative pyramidal neurons in this region **f**. Individual analysis of male **g** and female **h** populations also yielded no effect on the average firing rate of this region. *n* = 37–86 cells per group

To verify viral expression (by GFP reporter fluorescent), a subset of rats was transcardially perfused with saline (150 mL), followed by formaldehyde (150 mL; 4% w/v in phosphate-buffered saline (PBS)) at the cessation of all experiments. Rats were then quickly decapitated, and brains removed, post-fixed for at least 24 h (4% w/v formaldehyde in PBS) and cryoprotected (20% w/v sucrose in PBS) until saturated. Coronal sections (50 µm) of the vHipp and mPFC were cut using a cryostat (Leica, Buffalo Grove, UL, USA). Brain slices were mounted, and cover slipped with ProLong gold anti-fade reagent (120 µL). Slices were visualized on an OlympusIX81 motorized inverted confocal microscope and FV10-ASW software and enhanced using ImageJ (Fig. 1a, e).

### Analysis

Data are represented as the mean ± s.e.m. with *n* values representing the number of animals per experimental group, unless otherwise stated. Electrophysiological analysis was performed with commercially available computer software (LabChart version 7.1; ADInstruments, Chalgrove, Oxfordshire, UK). Statistics were calculated using SigmaPlot (Systat Software Inc.; Chicago, IL, USA). Data were analyzed by one-way ANOVA, one-way ANOVA on ranks or two-way ANOVA and the Holm-Sidak post-hoc test, with significance determined at *p* < 0.05.

### Materials

GIPZ Mouse Pvalb shRNA (Item #: VGM5520–200402107), GIPZ Mouse Sst shRNA (Item #: VGM5520–200337513) and GIPZ non-silencing lentiviral shRNA Control (Item #: RHS4348) were purchased from GE Healthcare (Lafayette, CO, USA). Chloral hydrate and Anti-Mouse IgG (whole molecule)- Peroxidase antibody produced in goat (A4416) was sourced from Sigma-Aldrich (St. Louis, MO, USA). ProLong® Gold antifade reagent (P36930) was purchased from Life Technologies (Carlsbad, CA, USA). Restore™ Western Blot Stripping Buffer (21059) and Pierce™ ECL Western Blotting Substrate (32106) were purchased from Thermo Fisher Scientific (Waltham, MA, USA). Any kD™ Mini-PROTEAN® TGX™ Precast Protein Gels (#4569035), Trans-Blot® Turbo™ Mini PVDF Transfer Pack (#1704156) and 2× Laemmli Sample Buffer (161–0737) were purchased from BioRad (Hercules, CA, USA). Anti-GAD56 + GAD67 antibody (ab49832), Goat Anti-Rabbit IgG H&L (HRP) (ab6721) and Anti-GAPDH antibody [mABcam 9484] – Loading Control (ab9484) were purchased from Abcam (Cambridge, MA, USA). All other chemicals and reagents were of either analytical or laboratory grade and purchased from standard suppliers.

## Results

### In vivo electrophysiology: Firing rate of putative pyramidal neurons in the vHipp

Aberrant activity in the vHipp has been observed in rodent models of schizophrenia. The average firing rate of putative pyramidal neurons in the vHipp of control rats (*n* = 88 neurons; 0.53 ± 0.05 Hz) is consistent with what has been previously demonstrated^21^. Rats with vHipp knockdown of PV (*n* = 79 neurons; 0.88 ± 0.06 Hz) or SST (*n* = 87 neurons; 0.73 ± 0.05 Hz) exhibited a significant increase in average firing rate (Fig. 1b; Kruskal–Wallis one-way ANOVA on ranks; H = 19.02; *P* < 0.001; Dunn’s Method; PV: Q = 4.30; *P* < 0.05; SST: Q = 2.73; *P* < 0.05). Interestingly, when male (*n* = 39 neurons; 1.02 ± 0.0 9 Hz; Fig. 1c) and female (*n* = 40 neurons; 0.74 ± 0.08 Hz; Fig. 1d) populations are analyzed separately, a significant increase in the firing rate was only observed for PV knockdown (Kruskal–Wallis one way ANOVA on ranks; Dunn’s Method; Males: H = 12.35; *P* = 0.002; Q = 3.48; *P* < 0.05; Females: H = 6.33; *P* = 0.04; Q = 2.46; *P* < 0.05) when compared to control rats (Males: *n* = 41 neurons; 0.62 ± 0.08 Hz; Females: *n* = 47 neurons; 0.45 ± 0.05 Hz).

### In vivo electrophysiology: Firing rate of putative pyramidal neurons in the mPFC

We measured the effects of PV and SST knockdown on mPFC putative pyramidal neuron activity and did not observe any significant differences between control (*n* = 83 neurons; 1.20 ± 0.15 Hz; Fig. 1f) and PV (*n* = 84 neurons; 1.76 ± 0.27 Hz) or SST (*n* = 86 neurons; 1.63 ± 0.28 Hz) knockdown groups. Similarly, there were no significant differences between any of the groups in male (control: *n* = 46 neurons; 0.99 ± 0.17 Hz; PV: *n* = 44 neurons; 1.23 ± 0.24 Hz; SST: *n* = 41 neurons; 1.92 ± 0.44 Hz; Fig. 1g) and female (control: *n* = 37 neurons; 1.46 ± 0.27 Hz; PV: *n* = 40 neurons; 2.35 ± 0.48 Hz; SST: *n* = 45 neurons; 1.38 ± 0.34 Hz; Fig. 1h) populations.

### In vivo electrophysiology: dopamine neuron activity

**Fig. 2:**
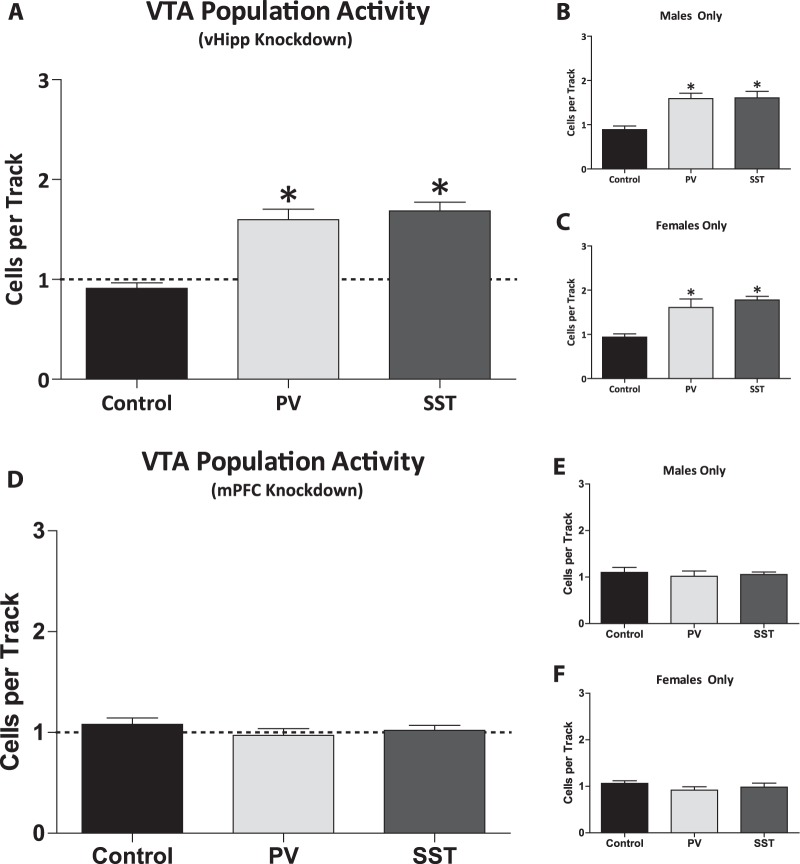
Parvalbumin or somatostatin expression selectively knocked down in the ventral hippocampus significantly increased dopamine neuron population activity. Dopamine neuron population activity was significantly increased in rats with parvalbumin (PV) or somatostatin (SST) expression selectively knocked down in the ventral hippocampus (vHipp) **a**. The same was observed in male **b** and female **c** populations when analyzed separately. *n* = 6–13 rats per group. *P < 0.05. In contrast, dopamine neuron activity was not altered by selective regional knockdown of PV or SST expression in the medial prefrontal cortex (mPFC) **d**. Separating male **e** and female **f** populations yielded the same result of no change in population activity of dopamine neurons. *n* = 6–13 rats per group

Increases in dopamine neuron population activity are consistently observed in various rodent models of schizophrenia. Similarly, selective regional knockdown of PV (*n* = 13 rats; 1.56 ± 0.10 cells/track) or SST (*n* = 13 rats; 1.67 ± 0.09 cells/track) in the vHipp lead to a significant increase in VTA dopamine neuron population activity (one-way ANOVA; *P* < 0.001; F_(2,38)_ = 25.48; Fig. 2a) when compared to control rats (*n* = 13 rats; 0.91 ± 0.05 cells/track; Holm–Sidak; PV: *t* = 5.78; *P* < 0.001; SST: *t* = 6.52; *P* < 0.001). No significant differences were observed between these groups in the average firing rates (control: n = 72 cells; 3.93 ± 0.28 Hz; PV: *n* = 115 cells; 3.66 ± 0.23 Hz; SST: *n* = 128; 3.90 ± 0.23 Hz) or percent burst firing (control: *n* = 72 cells; 39.48 ± 2.99%; PV: *n* = 115 cells; 41.47 ± 2.53%; SST: *n* = 128 cells; 39.33 ± 2.57%). Similar results were observed in both male and female rats (Fig. 2b, c).

In contrast to the results obtained in the vHipp, selective regional knockdown of PV (*n* = 13 rats; 0.97 ± 0.07 cells/track) or SST (*n* = 13 rats; 1.02 ± 0.05 cells/track) in the mPFC did not produce any significant differences in VTA dopamine neuron population activity (control: *n* = 13 rats; 1.08 ± 0.0.6 cells/track; Fig. 2d). No significant difference were observed between these groups in the firing rates (control: *n* = 83 cells; 4.03 ± 0.23 Hz; PV: *n* = 75 cells; 3.57 ± 0.25 Hz; SST: *n* = 79 cells; 3.35 ± 0.24 Hz) or percent burst firing (control: *n* = 83 cells; 38.46 ± 2.87%; PV: *n* = 75 cells; 34.20 ± 3.15%; SST: *n* = 79 cells; 38.21 ± 2.90%). Again, similar results were observed in both male and female rats (Fig. 2e, f)

### Amphetamine-induced locomotor response

**Fig. 3:**
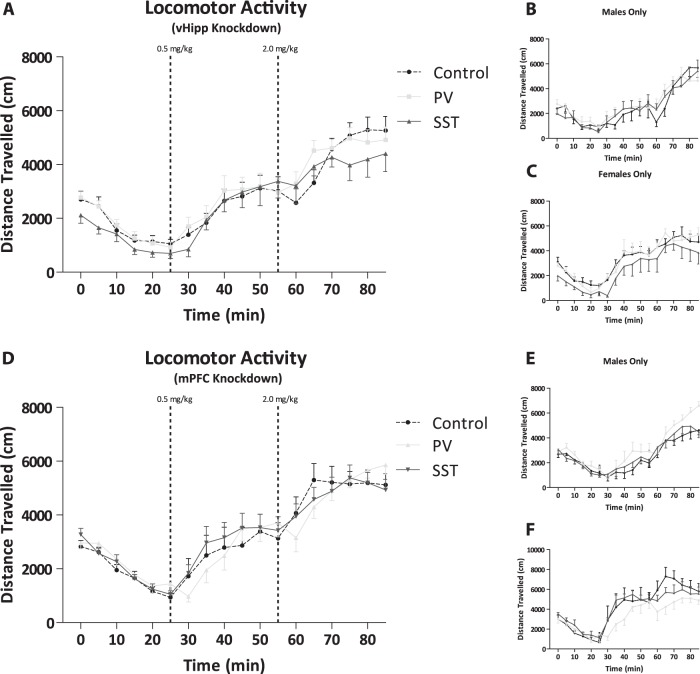
Amphetamine-induced locomotor response was not affected by selective regional knock down of parvalbumin or somatostatin in either region. Amphetamine-induced locomotor response was not affected by selective regional knock down of parvalbumin (PV) or somatostatin (SST) in the ventral hippocampus (vHipp) or medial prefrontal cortex (mPFC) in a population of male and female rats combined **a**, **d**. Subpopulations of male and female rats were also not affected by selective regional knockdown of PV or SST in the vHipp or mPFC **b**, **c**, **e**, **f**. *n* = 6–14 rats per group

Positive symptoms associated with schizophrenia can be modeled in rodents by measuring the distance traveled following exposure to psychostimulants, such as amphetamine. We did not observe any differences in rats with selective regional knock down of PV or SST in either the vHipp (Fig. 3a) or mPFC (Fig. 3d) when compared to their respective controls (*n* = 14 rats per group). Further analysis of these groups isolating just the male (Fig. 3b, e; *n* = 7–8 rats per group) or female (Fig. 3c, f; *n* = 6 rats per group) subjects yielded the same result of no significant differences in both the vHipp and mPFC respectively.

### Social interaction

**Fig. 4:**
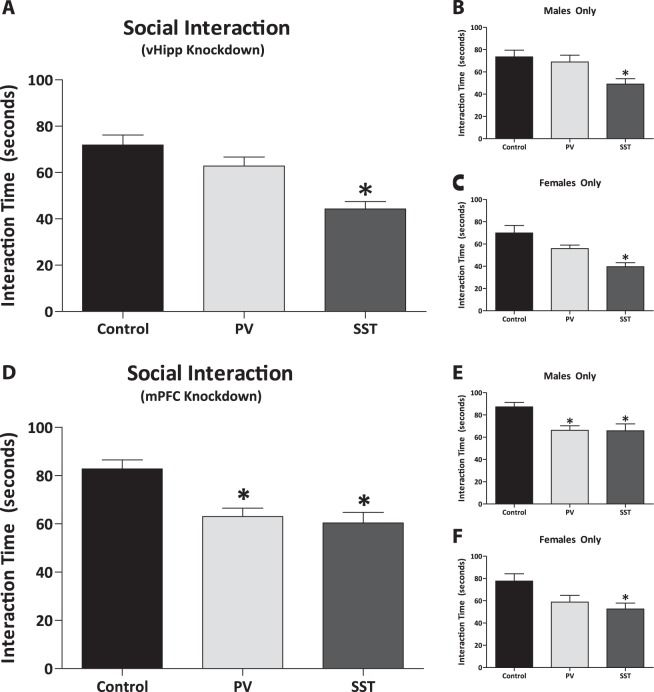
Social interaction is decreased by selective regional knock down of somatostatin (SST) expression in the ventral hippocampus (vHipp) and not by parvalbumin (PV) in male and female rats combined. **a**. Subpopulations of male **b** and female **c** rats were also affected by only SST expression knock down in the vHipp, and not by PV expression knockdown of this region. In the medial prefrontal cortex (mPFC), both PV and SST expression knock down results in a significant decrease in the time spent interacting in mixed population (males and females, **d**). The same behavioral pattern is observed in a male only population **e**; however, female rats are only affected by selective regional knockdown of SST, and not PV expression in the mPFC **f**. *n* = 6–18 rats per group. *P < 0.05

Negative symptoms can be correlated in rodents by measuring the interaction time between a test rat and stimulus rat, thus we performed a social interaction behavioral assay on rats that had PV or SST selectively knocked down in the vHipp or mPFC. We observed a significant decrease in the time spent interacting in male and female rats combined that had a selective knockdown of SST (*n* = 18 rats; 44.31 ± 3.19 s) in the vHipp when compared to controls (Fig. 4a; *n* = 15 rats; 71.87 ± 4.3 s; Kruskal–Wallis one way ANOVA on Ranks; H = 20.93; *P* < 0.001; Dunn’s Method; Q = 4.42; *P* < 0.05). No significant differences were observed in rats with selective regional knockdown of PV in the vHipp (*n* = 15 rats; 62.83 ± 3.89 s). Similarly, when male (Fig. 4b; control: *n* = 8 rats; 73.56 ± 6.01 s; SST: *n* = 9 rats; 49.0 ± 4.97 s) and female (Fig. 4c; control: *n* = 7 rats; 69.93 ± 6.69 s; SST: *n* = 9 rats; 39.61 ± 3.59 s) populations were separated, both groups displayed a significant decrease in the time spent interacting only when SST (males: Kruskal–Wallis one way ANOVA on Ranks; H = 8.31; *P* = 0.02; Dunn’s Method; Q = 2.64; *P* < 0.05; females: one way ANOVA; F_(2,22)_ = 11.34; *P* < 0.001; Holm–Sidak; *t* = 4.73; *P* < 0.001) was selectively knocked down. No differences were observed in the groups with PV selectively knocked down in the vHipp (males: *n* = 8 rats; 68.94 ± 6.11 s; females: *n* = 7 rats; 55.86 ± 3.21 s).

Rats that had PV or SST selectively knocked down in the mPFC displayed a significant decrease in the time spent interacting in male and female rat populations combined, that had a selective knockdown of PV (*n* = 14 rats; 63.00 ± 3.52 s) and SST (*n* = 17 rats; 60.38 ± 4.42 s) in the mPFC when compared to controls (Fig. 4d; *n* = 15 rats; 82.77 ± 3.80 s; one way ANOVA; F_(2, 45)_ = 9.33; *P* < 0.001; Holm–Sidak post-hoc; PV: *t* = 3.39; *P* = 0.003; SST: *t* = 4.03; *P* < 0.001).

### Attentional set-shifting

**Fig. 5:**
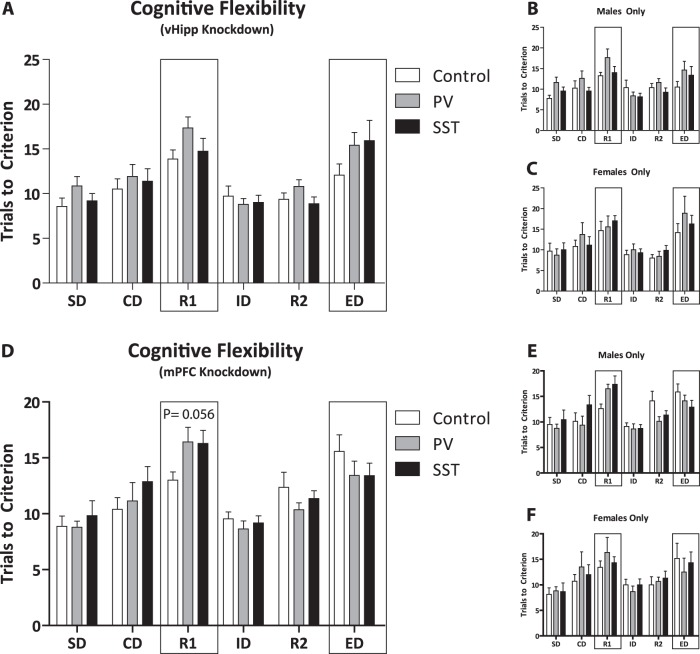
Reversal learning, and extra-dimensional set-shifting tasks are not affected by selective regional knock down of parvalbumin or somatostatin in either region. Reversal learning, and extra-dimensional set-shifting tasks are not affected by selective regional knock down of parvalbumin (PV) or somatostatin (SST) in the ventral hippocampus (vHipp) or medial prefrontal cortex (mPFC) in a population of male and female rats combined **a**, **d**. Subpopulations of male and female rats displayed a trend (*p* = 0.056) to increased trials to criterion in the reversal learning stage, suggesting a possible deficit in cognitive flexibility; however, this was primarily prevalent in the male rats as females were not affected by selective regional knockdown of PV or SST in the vHipp or mPFC **b**, **c**, **e**, **f**. *n* = 6–15 rats per group

Cognitive deficits are commonly observed in individuals with schizophrenia and can be evaluated in rodents using the attentional set-shifting assay. Deficits are observed in rodent models of the disease in specific portions of the test, namely reversal learning and extra-dimensional set-shifting^27–29^, as measured by the number of trials to reach criterion (TTC). A mixed population with male and female rats did not display any deficits in reversal learning (Fig. 5a; control: *n* = 14 rats; 13.86 ± 1.04 TTC; PV: *n* = 15 rats; 17.33 ± 1.24 TTC; SST: *n* = 15 rats; 14.73 ± 1.44 TTC) or extra-dimensional set shifting (control: 12.07 ± 2.65 TTC; PV: 15.4 ± 1.45 TTC; SST: 15.93 ± 1.34 TTC) with PV or SST expression knockdown in the vHipp; however, although not significant, there appears to be a trend towards a deficit in reversal learning, as indicated by an increase in the number of trials to reach criterion, in the PV knock down group.

In contrast, mPFC knockdown of PV or SST expression appeared to cause a deficit (*p* = 0.056) in reversal learning with both PV and SST expression decreases in the mPFC causing an increase in TTC, indicative of a deficit in cognitive flexibility (Fig. 5d; control: *n* = 15 rats; 13.0 ± 0.74 TTC; PV: *n* = 14 rats; 16.43 ± 1.30 TTC; SST: *n* = 17 rats; 16.29 ± 1.17 TTC; one-way ANOVA; *P* = 0.056). Further analysis of male (Fig. 5e; control: *n* = 8 rats; 12.63 ± 0.89 TTC; PV: *n* = 8 rats; 16.5 ± 0.89 TTC; SST: *n* = 11 rats; 17.36 ± 1.64 TTC) and female (Fig. 5f; control: *n* = 7 rats; 13.43 ± 1.64; PV: *n* = 6 rats; 16.33 ± 2.96; SST: *n* = 6 rats; 14.33 ± 1.17 TTC) populations suggested no change in reversal learning in either group. No deficits were observed in extra-dimension set-shifting (control: *n* = 14 rats; 15.57 ± 1.49 TTC; PV: *n* = 14 rats; 13.43 ± 1.29 TTC; SST: *n* = 17 rats; 13.41 ± 1.10 TTC).

### Western Blot

We did not detect any significant differences in the protein expression of GAD 65/67 from the vHipp knockdown rats (PV knockdown = 109.20 ± 9.24% control; *n* = 12; SST knockdown = 100.25 ± 22.08% control; *n* = 10) or mPFC knockdown rats (PV knockdown = 112.83 ± 22.13% control; *n* = 6; SST knockdown = 104.05 ± 16.05% control; *n* = 4). It should be noted, however, that western blot analysis of the region may not reflect GAD expression in the transfected cells, as the homogenate contains additional interneuron subtypes, as well as un-transfected PV/SST neurons.

## Discussion

Schizophrenia is marked by three classical symptoms domains including: positive and negative symptoms and cognitive dysfunction. Available antipsychotic pharmacotherapies treat the positive associated symptoms, but are often discontinued due to ineffectiveness or undesirable side-effects^30^. Additionally, these drugs have little to no effect on the negative symptoms and cognitive impairments experience by individuals with schizophrenia^30^. As mentioned previously, positive symptoms of the disease are attributed to dysfunction of the mesolimbic dopamine system, commonly observed in patients;^15,16^ however, no evidence of a primary pathology in the dopamine neurons themselves exists^17^. Thus, it is believed that it is in fact aberrant regulation of the dopamine system, by the anterior hippocampus, that drives symptoms of psychosis. Clinical studies have revealed hyperactivity in the anterior hippocampus at rest in individuals with schizophrenia^18^, which is also present in the vHipp of rodent models^19,21^. Additionally, the prefrontal cortex has been implicated as a key brain region involved in cognitive flexibility and the likely site of pathology for negative symptoms^31–34^. Alterations in prefrontal cortical function is associated with cognitive dysfunction in individuals with schizophrenia^35,36^ and rodent models^37,38^. The common pathology that links aberrant hippocampal and prefrontal cortical activity is a deficit in GABAergic neurotransmission, commonly observed in individuals with schizophrenia^1,4,39^. Thus, it is possible that intrinsic GABAergic signaling deficits may be the origin of the observed hippocampal hyperactivity and cognitive impairments in individuals with schizophrenia. In this study we examined the role of two distinct subpopulations of interneurons (PV and SST) and correlated changes in neuronal activity with behaviors associated with positive and negative symptoms, as well as cognitive flexibility.

Studies performed post-mortem in human brain tissue report regional decreases of interneurons expressing the calcium binding protein, PV and the neurotransmitter, SST in the hippocampus and frontal cortex of patients with schizophrenia^4,9,40–42^. Similarly, rodent models of the disease display specific reductions in PV interneuron density in analogous regions (vHipp and mPFC)independent of total interneuron number or changes in glutamic acid decarboxylase 67 (GAD67)^12,43^. PV positive interneurons are fast-spiking and perisomatic, ideally situated to regulate the activity of pyramidal neurons^44^. Pre-clinical data support the notion that a reduction in PV interneuron function is indeed correlated with altered vHipp and mPFC activation during task performance^12^. SST positive cell loss has been observed in the limbic and cortical brain regions of patients with schizophrenia^11,45–47^. This is important as neuropeptides, such as SST, play a vital role in the inhibitory control of pyramidal neurons in both the vHipp and mPFC^1,48,49^. Moreover, impairments in cognitive function are observed when cortical assemblies are not properly activated^12^. We used commercially available high-titer lentivirus particles containing GIPZ vectors expressing shRNA targeting either PV (Fig. 1a) or SST (Fig. 1e), specifically in the vHipp or mPFC, and performed a series of behavioral and electrophysiological assays to determine the relative contributions of each interneuron subpopulation in the specified region.

Increasing evidence supports the idea that aberrant hippocampal activity may underlie the pathological increase in dopamine system function observed in schizophrenia patients and in rodent models. Indeed, we have previously demonstrated that with pharmacological^19^, surgical^27,50^, and cell-based modulation of vHipp activity^21,22^, we can reverse positive and negative symptoms, as well as cognitive deficits associated with schizophrenia. In the current study, we used in vivo electrophysiology to observe the firing rates of putative pyramidal neurons of the vHipp and mPFC in anesthetized rats. We found that selectively decreasing PV or SST mRNA expression in the vHipp of Sprague–Dawley rats (male and female rats combined) is sufficient to produce baseline hippocampal hyperactivity consistent with that observed in rodent models and individuals with schizophrenia (Fig. 1b). These data are consistent with a previous study performed in our laboratory where a reduction in vHipp PV expression was sufficient to increase vHipp activity^20^. The mechanism by which decreases in PV mRNA expression lead to hippocampal hyperactivity has yet to be elucidated. As mentioned previously, PV is a calcium binding protein, and as such, it can alter the decay of intracellular calcium without changing the amplitude of fast calcium transients^51^. Following high frequency stimulation, PV knockout mice display an enhanced facilitation of GABA release^52^, while a loss of PV expression does not change single inhibitory post-synaptic responses or paired-pulse modulation of IPSCs in the hippocampus of transgenic mice^52^. This apparent discrepancy may reflect differences between developmental knockout and shRNA-mediated knockdown in adulthood. Indeed, PV has been suggested to protect against cell death in conditions such as epilepsy^53–55^. Thus, whether PV knockdown results in cell death and/or aberrant cell function has not been conclusively demonstrated, however we believe that the net result is a decrease in GABAergic neurotransmission, as demonstrated by an increase in vHipp activity.

Baseline firing rates in the mPFC did not appear to be affected by decreases in PV or SST mRNA expression in any of the groups. Previously reported data in a rodent model with disruptions in GABAergic signaling in the mPFC, also reported no changes in anesthetized rats, but did see a significant decrease with the addition of PV positive interneuron cell transplants in the mPFC^56^. It is possible that differential sensitivities of mPFC neurons to the effects of chloral hydrate anesthesia may have confounded the interpretation of these results, as it has been demonstrated to modify the pattern of discharge for individual neurons in the cortical areas^57,58^. This anesthetic was specifically chosen because it has been shown not to alter dopamine neuron activity^59^. Additionally, mPFC activity was examined at rest and does not reflect changes in prefrontal cortex function observed during cognitive function. Robust deficits have been observed during task performance, where patients fail to adequately activate the PFC^60,61^. Preclinical data in rodent models of the disease report a failure to engage the mPFC during a behavioral paradigm^12^.

Based on our previous studies, we predict that the increased vHipp activity in rodents with decreased vHipp PV and SST mRNA expression, likely induces downstream changes in VTA dopamine neuron activity. Indeed, we now report that males, females and the combined male and female group with PV and SST knocked down in the vHipp all display a significant increase in VTA dopamine neuron population activity. These data are consistent with numerous rodent models of the disease that also display increased population activity of VTA dopamine neurons^19,23,62,63^. Furthermore, the aberrant vHipp activity and subsequent increase in population activity did not alter dopamine neuron firing rate or percent of action potentials firing in a burst, also consistent with what has been previously documented. Given that the mPFC also plays a role in the regulation of dopamine neuron activity and that the pattern of coordinated activity in the cortex is a critical component in determining dopamine system output^64^, we examined whether knocking down PV or SST expression in this region was sufficient to modulate VTA dopamine neuron activity. Interestingly, the increase in dopamine neuron population activity was not altered by a decrease in PV or SST expression in the mPFC. These data are consistent with the hypothesis that aberrant dopamine system function, which likely contributes to positive symptoms of schizophrenia, is driven by hyperactivity of the hippocampus that is largely independent of the mPFC (at rest). Indeed, it was previously demonstrated that increases in VTA population activity, observed following vHipp activation, is dependent on glutamatergic activity in the nucleus accumbens and not altered by blockade of the mPFC^65^.

Measuring symptoms such as hallucinations and delusions is impossible to assess in rodents; however, we can measure the enhanced response to psychostimulants observed in patients. Previous studies in rodent models of the disease report an enhanced response to the locomotor-inducing effects of amphetamine;^20,21,27,50^ however, we were not able to reproduce this response in rats with PV or SST mRNA expression in the vHipp or mPFC. We expected the rats with an altered dopamine neuron activity present in response to a hyperactive hippocampus to display an increased sensitivity to amphetamine, however this was not the case. This may be associated, in part, with potential sex differences as a trend to increase locomotor activity in males was observed, consistent with our previous reports^20^, while females rats display enhanced sensitivity to amphetamine during specific stages of the estrous cycle^66^ and the stage of the estrous cycle was not tracked during this study.

To assess whether vHipp/mPFC knockdown of PV or SST alters behavior associated with negative symptoms, rats were tested in the social interaction paradigm. During this test, the amount of time the test animal spends engaged in active behavior (i.e., sniffing, following, approaching, climbing on or under, etc.) with an unfamiliar stimulus rat is observed. Social withdrawal is indeed a prominent symptom in individuals with schizophrenia and can dramatically decrease a patient’s quality of life^67–69^. Here we demonstrate that decreases in SST (but not PV) mRNA expression in vHipp was enough to cause a decrease in social interaction, while knockdown of both PV and SST in mPFC significantly decreased interaction time. Sex differences were not present in this behavioral task. These data add to the notion that alterations in mPFC function may contribute to negative symptoms, including social withdrawal. Indeed, a significant literature demonstrates the involvement of the mPFC in social behavior^70,71^.

Cognitive decline is not only common in schizophrenia but is also prevalent in many neuropsychiatric illnesses^72^. Therapies available for the treatment of cognitive decline, including deficits in working memory and cognitive flexibility, is a major unmet need for the treatment of schizophrenia. The mPFC has been consistently implicated in set-shifting, which can be measured in rodents using the attentional set-shifting assay^26^. Thus, we used this paradigm to examine the utility of PV or SST mRNA expression in the vHipp or mPFC in causing a deficit in reversal learning and extra-dimensional set shifting, which has been previously demonstrated in rodent models of the disease^22,27,28^. Knock down of PV and SST mRNA in the mPFC appeared to produce an increase in reversal learning. We have previously examined the role of SST and PV interneurons in the regulation of cognitive function by transplanting stem-cell derived PV and SST neuron transplants in the vHipp of a rodent model of schizophrenia. These studies reported a reversal of deficits in reversal learning with both PV and SST, while extra-dimensional set-shifting was ameliorated only with PV stem cell transplants^22^. Similarly, stem cell transplantation of PV interneurons into the mPFC can also reverse deficits in reversal learning^56^. These data support the hypothesis that PV and SST interneuron populations in the mPFC contribute to cognitive flexibility.

Taken together, we demonstrated that selective regional knock down (in the vHipp and mPFC) of PV or SST interneuron populations is sufficient to cause changes in the firing rates of local neuron population and downstream dopamine neuron population activity (relevant to positive symptoms), as well as behavioral deficits in negative symptoms and cognitive flexibility. Specifically, PV and SST knockdown in the vHipp contributes to aberrant dopamine neuron activity, thought to contribute to positive symptoms; whereas, targeting the mPFC produced deficits in social behavior and cognitive function. These results further demonstrate that deficits in GABAergic signaling may be a key pathology contributing to the neurophysiological and behavioral deficits observed in schizophrenia and suggest that an effective therapeutic strategy may be to restore interneuron regulation of patterned activity in the vHipp and mPFC.
